# Changes in N:P Supply Ratios Affect the Ecological Stoichiometry of a Toxic Cyanobacterium and Its Fungal Parasite

**DOI:** 10.3389/fmicb.2017.01015

**Published:** 2017-06-06

**Authors:** Thijs Frenken, Joren Wierenga, Alena S. Gsell, Ellen van Donk, Thomas Rohrlack, Dedmer B. Van de Waal

**Affiliations:** ^1^Department of Aquatic Ecology, Netherlands Institute of Ecology (NIOO-KNAW),Wageningen, Netherlands; ^2^Department of Biology, University of UtrechtUtrecht, Netherlands; ^3^Department of Plant and Environmental Sciences, Norwegian University of Life SciencesÅs, Norway

**Keywords:** nutrients, harmful algal blooms, plankton, Chytridiomycota, disease, pathogen, microcystin

## Abstract

Human activities have dramatically altered nutrient fluxes from the landscape into receiving waters. As a result, not only the concentration of nutrients in surface waters has increased, but also their elemental ratios have changed. Such shifts in resource supply ratios will alter autotroph stoichiometry, which may in turn have consequences for higher trophic levels, including parasites. Here, we hypothesize that parasite elemental composition will follow changes in the stoichiometry of its host, and that its reproductive success will decrease with host nutrient limitation. We tested this hypothesis by following the response of a host–parasite system to changes in nitrogen (N) and phosphorus (P) supply in a controlled laboratory experiment. To this end, we exposed a fungal parasite (the chytrid *Rhizophydium megarrhizum*) to its host (the freshwater cyanobacterium *Planktothrix rubescens*) under control, low N:P and high N:P conditions. Host N:P followed treatment conditions, with a decreased N:P ratio under low N:P supply, and an increased N:P ratio under high N:P supply, as compared to the control. Shifts in host N:P stoichiometry were reflected in the parasite stoichiometry. Furthermore, at low N:P supply, host intracellular microcystin concentration was lowered as compared to high N:P supply. In contrast to our hypothesis, zoospore production decreased at low N:P and increased at high N:P ratio as compared to the control. These findings suggest that fungal parasites have a relatively high N, but low P requirement. Furthermore, zoospore elemental content, and thereby presumably their size, decreased at high N:P ratios. From these results we hypothesize that fungal parasites may exhibit a trade-off between zoospore size and production. Since zooplankton can graze on chytrid zoospores, changes in parasite production, stoichiometry and cell size may have implications for aquatic food web dynamics.

## Introduction

Human activities have substantially increased the flux of nutrients from land into receiving waters (Smith, 2003). This nutrient enrichment enhances aquatic primary production, and may lead to dramatic changes in the composition and structure of aquatic food webs (Schindler and Fee, 1974; Smith et al., 2006). Specifically, an increased nutrient supply might promote development of harmful cyanobacterial blooms (Paerl et al., 2001, 2011; Smith and Schindler, 2009). Although nutrient loading has increased, primary production in aquatic ecosystems is often still limited by nitrogen (N) and/or phosphorus (P) (Elser et al., 2007; Bracken et al., 2014). This may be a result of an imbalanced nutrient supply (Carpenter et al., 1996; Sterner et al., 2007), as well as an increased nutrient demand associated to high phytoplankton densities (Carpenter et al., 1996). Nutrient limitation will alter the elemental composition of phytoplankton, and may specifically increase carbon:nutrient ratios (Sterner and Elser, 2002). As a consequence, nutritional quality of the phytoplankton decreases, thereby possibly constraining higher trophic levels (Sterner and Elser, 2002; Hessen et al., 2013). This may particularly apply to parasites that solely depend on their host as a food source (Smith, 2007).

Fungal parasites are very common pathogens infecting phytoplankton (Gerphagnon et al., 2015), which represent an important but yet overlooked ecological driving force in aquatic food web dynamics (Sime-Ngando, 2012). These parasites, belonging to the phylum Chytridiomycota and often referred to as chytrids, are host specific zoosporic fungi that can parasitize on phytoplankton and completely rely on their host to obtain energy and nutrients leading to death of the host (Sparrow, 1960, 1968; Barr, 2001). Thereby, they play an important role in natural aquatic ecosystems, in which chytrids can significantly change phytoplankton abundance and seasonal succession (Reynolds, 1973; Van Donk, 1984, 1989). Additionally, the free swimming stage of the chytrids (i.e., zoospores) may provide higher trophic levels with an alternative food source during blooms of large inedible diatoms (Kagami et al., 2007; Frenken et al., 2016) or cyanobacteria (Agha et al., 2016). Earlier work indicates that zoospores might find their host by chemotaxis (Muehlstein et al., 1988), and penetrate host cells using a rhizoidal system through which nourishment is conveyed to the zoospore (Van Donk and Ringelberg, 1983). After infection, the spore forms a sessile stage (i.e., sporangium) in which new zoospores (up to 60) are produced (Canter and Lund, 1951; Sparrow, 1960).

Chytrid zoospores generally contain a relatively high amount of nucleic acids that are particularly rich in P, but also contain substantial amounts of lipids, including fatty acids and sterols, which are rich in carbon (Barr and Hadland-Hartmann, 1978; Beakes et al., 1988, 1993; Elser et al., 1996; Kagami et al., 2007). Chytrids thus seem to have high P demands, as has been indicated by their low C:P as compared to their host (Kagami et al., 2007). As a consequence, limitation by P may affect a chytrid more than its host. Chytrid infections were indeed shown to be affected by host P limitation. More specifically, chytrid growth rate and the number of zoospores per sporangium decreased, and, as a function of lower host growth rate, zoospore loss as well as searching time increased, as compared to non-limited conditions (Bruning, 1991). If, however, P limitation impedes algal growth to a greater extent than that of the chytrid, epidemics may still occur (Bruning and Ringelberg, 1987; Bruning, 1991).

Nutrient limitation not only alters growth and reproduction of the parasite, it may also affect host defense. Freshwater cyanobacteria produce a wide range of oligopeptides including toxic microcystins (MC) (Welker and Von Döhren, 2006), which have been associated to chytrid defense (Rohrlack et al., 2013). These oligopeptides are N rich compounds, and their synthesis is typically constrained under N limitation (Van de Waal et al., 2010, 2014). Thus, during low N:P conditions, host defense may be reduced and thereby facilitate chytrid infections. In contrast, cellular N may accumulate under high N:P conditions and thereby enhance host defense. Limitation by N and P may thus have contrasting effects on chytrid infections of cyanobacteria. We hypothesized that parasite elemental composition will follow changes in the stoichiometry of its host, and that its reproductive success will decrease with host nutrient limitation. To test this hypothesis, we exposed the cyanobacterium *Planktothrix rubescens* to its chytrid *Rhizophydium megarrhizum* under control, low N and low P conditions, leading to a range of host N:P ratios. We predict that infections will decrease with increasing host N:P, as the availability of P for chytrid nutrition will decrease and the host defense by oligopeptides will increase.

## Materials and Methods

### Description of Test Organisms

In this study the filamentous cyanobacterial host *P. rubescens* NIVA-CYA97/1 was used in combination with one of its parasites, the chytrid Chy-Lys2009 (photo provided in the Supplementary Material). This chytrid possesses identical morphological characteristics and infection patterns in agreement with *R. megarrhizum* described earlier by Canter and Lund (1951). More information on host specificity and virulence of the chytrid can be found in Sønstebø and Rohrlack (2011) and Rohrlack et al. (2013). All cultures used in this study were monoclonal and non-axenic.

### Culture Maintenance

The *Planktothrix* and the chytrid Chy-Lys2009 cultures were grown in a temperature and light controlled incubator (Snijders Labs, Tilburg, The Netherlands) at 5 μmol photons m^-2^ s^-1^ in a 14:10 light:dark cycle, at 24 and 16°C, respectively. The applied low light levels resemble the conditions where the tested *Planktothrix* species was isolated, i.e., in the vicinity of the thermocline. All cultures were maintained in exponential growth in batch using 100 mL Erlenmeyer flasks with 50 mL suspension. Every other week, *Planktothrix* cultures were diluted using WC-medium (Guillard and Lorenzen, 1972) and chytrid cultures were diluted using host culture and WC-medium to 1/10 (v/v). Additionally, Erlenmeyer flasks were shaken every other day to prevent aggregation. The chytrid cultures were visually inspected for infection patterns and contaminations at least once a week.

### Description of the Experiment

#### Culture Acclimatization and Inoculation

Prior to the experiment, *Planktothrix* was grown at 16°C on WC-medium at three distinct N:P supply ratios by modifying standard NO_3_^-^ and PO_4_^3-^ concentrations of 1000 and 50 μmol L^-1^ (N:P = 20) as control, to 200 and 50 μmol L^-1^ (N:P = 4) as the low N:P treatment, and 1000 and 10 μmol L^-1^ (N:P = 100) as the high N:P treatment. Cultures were acclimatized for about 18 generations to the distinct nutrient conditions by three consecutive transfers at late exponential phase. During each transfer, i.e., after each 7 days period, cultures were diluted back to half of maximum biovolume reached in order to maintain nutrient limited conditions. After acclimatization, *Planktothrix* was first grown without chytrids (unexposed treatment, 4 replicates per nutrient treatment, 12 experimental units) to late stationary phase to obtain uninfected host growth rates, stoichiometry and toxin composition. Subsequently, the host cultures were then pooled by nutrient treatment and used to inoculate the chytrid exposed treatments (4 replicates per nutrient treatment, 12 experimental units). At the start of the chytrid exposed treatment, *Planktothrix* cultures were inoculated together with a zoospore suspension that was obtained from a highly infected *Planktothrix* culture (with 58% Chy-Lys2009 infected filaments) by sieving gently over a 30 μm and a subsequent 5 μm nylon mesh to remove host cells, while collecting zoospores that have a typical size range of 2.5–3.5 μm (Sparrow, 1960). This zoospore suspension was washed with N and P free WC-medium and concentrated on a 1.2 μm cellulose acetate membrane filter (Whatman, Maidstone, United Kingdom), and used to inoculate to a final density of 18 zoospores mL^-1^. The chytrid exposed cultures were grown for 7 days to obtain host and parasite growth rates, host and parasite stoichiometry, parasite zoospore production and toxin composition of parasite exposed host. Each treatment was performed in 500 mL Erlenmeyer flasks with 300 mL of culture.

#### Host and Parasite Quantification

During the experiment, cultures were sampled daily to determine biovolume using a CASY Cell Counter (Schärfe System GmbH, Reutlingen, Germany). Next, at least 5 mL of culture suspension was fixed with alkaline Lugol’s iodine solution to a final concentration of 1.2% (v/v) and stored in the dark at room temperature. Prevalence of infected filaments was counted in duplicate (technical replicate) for each biological replicate within 2 weeks after the experiment by inspecting at least 50 filaments. Additionally, during the infection treatment, the number of free swimming zoospores was counted daily in duplicate, also for each biological replicate, in at least 15 fields of view (FOV) in fresh cultures. All microscopic counting was performed using a magnification of 200× on an inverted microscope (DMI 4000B, Leica Microsystems CMS GmbH, Mannheim, Germany). Cultures were harvested at the early stationary phase for the analyses of dissolved inorganic nutrients, elemental composition of the host and parasite, and the MC contents and composition of the host.

#### Elemental Analyses

Particulate organic carbon (C), N and P were determined in duplicate by collecting 5–15 mL of seston on a prewashed GF/F filter (Whatman, Maidstone, United Kingdom) applying gentle filtration (<1–2 psi). Filters were dried overnight at 60°C, and stored in a desiccator in the dark. For C and N analyses, a subsample (22%) of every filter was taken by a hole puncher, folded into a tin cup and analyzed on a FLASH 2000 organic elemental analyzer (Brechbueler Incorporated, Interscience B.V., Breda, The Netherlands). Particulate organic P was analyzed (Eaton, 2005) by first combusting the remainder of the filter (78%) for 30 min at 550°C in Pyrex glass tubes, followed by a digestion step with 2.5 mL persulfate (2.5%) for 30 min at 120°C. This digested solution was measured for PO_4_^3-^ on the QuAAtro39 AutoAnalyzer (SEAL Analytical Ltd, Southampton, United Kingdom) following Armstrong et al. (1967). During the infection treatment, particulate organic C, N and P were determined for the seston fraction as well as for the zoospores. For this purpose, 90–160 mL infected culture suspension was gently filtered twice over a 30 μm nylon mesh filter to remove the larger cyanobacterial filaments. Subsequently, the smaller filaments were removed by an additional filtration over a 5 μm nylon mesh, and zoospores in the filtrate were collected on a prewashed GF/F filter (Whatman GF/F, Maidstone, United Kingdom). Organic C, N and P on the filters were analyzed as described above.

#### Microcystin Analyses

##### Extractions

Samples for MC analyses were collected in duplicate by filtering 5–15 mL of culture over a GF/C filter (Whatman, Maidstone, United Kingdom) applying low pressure after which the filters were stored at -20°C. Filters were lyophilized overnight before performing three rounds of extractions at 60°C using 2.5 mL 75% methanol-25% Millipore water (v/v) in 8 mL Pyrex glass tubes. After drying the samples with N_2_, extracts were reconstituted in 900 μl methanol, filtered and centrifuged (Corning© Costar© Spin-X© polypropylene centrifuge tube filters with a 0.22 μm cellulose-acetate filter (Corning Inc., Corning, NY, United States) for 5 min at 16,000 ×*g* (Sigma 1-15P, Sigma Laborzentrifugen GmbH, Osterode am Harz, Germany). Filtrates were transferred to amber glass vials and analyzed by LC-MS/MS.

##### Analyses

Samples were analyzed for eight MC variants (dm-7-MC-RR, MC-RR, MC-YR, dm-7-MC-LR, MC-LR, MC-LY, MC-LW, and MC-LF). Calibration standards were obtained from the National Research Council (Ottawa, Canada) for dm-7-MC-LR, and from Enzo Life Sciences Inc. (Farmingdale, NY, United States) for the other variants. Measurements were performed on an Agilent 1260 LC and an Agilent 6460A QQQ (Agilent Technologies, Santa Clara, CA, United States). The compounds were separated on an Agilent Zorbax Eclipse XDB-C18 4.6 mm × 150 mm, 5 μm column using Millipore water with 0.1% formic acid (*v*/*v*, eluent A) and acetonitrile with 0.1% formic acid (*v*/*v*, eluent B). The elution program was set at 0–2 min 30% B, 6–12 min 90% B, with a linear increase of B between 2 and 6 min and a 5 min post run at 30% B. Sample injection volume was set at 10 μL, with a flow of 0.5 mL min^-1^ at a column temperature of 40°C. The LC-MS/MS was operated in positive mode with an ESI source, nitrogen was used as a drying, sheath and collision gas. For each compound, two transitions were monitored in MRM mode: *m/z* 491.3 to *m/z* 135.1 and *m/z* 981.5 to *m/z* 135.2 (dm-7-MC-LR, ratio between product ions 17%), *m/z* 498.3 to *m/z* 135.1 and *m/z* 995.6 to *m/z* 135.1 (MC-LR, ratio between product ions 16%). This protocol is based on the protocol earlier described by Faassen and Lürling (2013).

### Data Analyses

Host population growth and zoospore production rates were calculated according to μ = ln (B_n+t_/B_n_)/t. In which μ is the maximum specific growth rate, *B*_n_ is the initial population density of non-infected or infected host (biovolume), or zoospores (counts), *B*_n+t_ is the final population density of these variables over time *t* in the exponential growth phase. Infected biomass was calculated by multiplying the proportion of infected filaments with biovolume. Zoospore production efficiency was calculated as the number of zoospores produced per infected host biovolume.

Host maximum specific growth rate, zoospore production rate, seston stoichiometry of the host and parasite and MC content of the host were tested for normality and equal variance using the Shapiro–Wilk and Brown-Forsythe tests, respectively. Data were transformed, log or reciprocal, if this improved normality. Host growth rates, zoospore production rate, seston and zoospore stoichiometry and host MC content were analyzed to test for effects of nutrient supply by performing a one-way ANOVA. Pairwise comparisons were conducted using the Holm-Sidak test (Sidak, 1967). The strength and direction of associations between variables were assessed by Pearson product-moment correlations. All analyses were performed using SigmaPlot version 13 (Systat Software Inc., London, United Kingdom). Detailed output of the different statistical tests can be found in the Supplementary Material.

## Results

### Host Growth and Biovolume Build-Up

**FIGURE 1:**
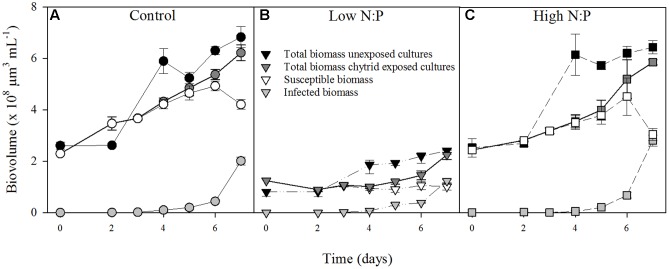
Biovolumes of *Planktothrix* in the in the cultures with and without parasite exposure in the control **(A)**, low N:P **(B)** and high N:P **(C)** treatments. Symbols represent mean ± standard error (*n* = 4).

In the absence of the parasite, *Planktothrix* population growth rates were comparable in all treatments (**Table 1**), with replicates ranging between 0.30 and 0.59 d^-1^. In the presence of the parasite, nutrient supply also had no clear effect on net population growth rate of the total biovolume (**Table 1**). After 4 days of infection, infected host biomass increased at the expense of susceptible host biomass (**Figure 1**). The total *Planktothrix* biomass build-up after 4–7 days was lower in the chytrid exposed cultures than in the unexposed cultures. The rate at which the infected biomass increased was highest under a high N:P supply, while it did not differ between the control and low N:P treatment (**Table 1**).

**Table 1:** *Planktothrix* maximum net population growth rates (d^-1^) of the different biomass fractions in the unexposed and chytrid exposed cultures.

	Unexposed	Exposed		
Treatment	Total	Total	Susceptible	Infected
Low N:P	0.42 ± 0.05^a^	0.06 ± 0.05^a^	-0.05 ± 0.02^a^	0.94 ± 0.09^a^
Control	0.40 ± 0.04^a^	0.11 ± 0.04^a^	0.10 ± 0.03^b^	0.92 ± 0.09^a^
High N:P	0.40 ± 0.06^a^	0.11 ± 0.03^a^	0.10 ± 0.03^b^	1.34 ± 0.09^b^

*Superscript letters denote significant differences between nutrient treatments based on One-way ANOVA and post hoc comparison of the means (α < 0.05).*

### Elemental Composition

**FIGURE 2:**
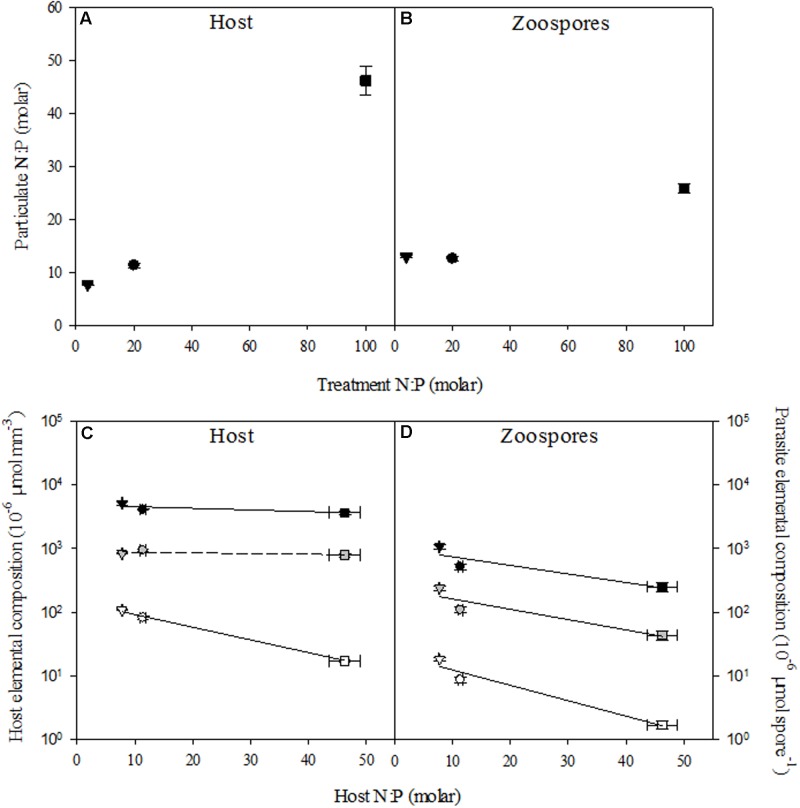
N:P ratios of uninfected host **(A)** and zoospores **(B)**, and elemental content of the host **(C)** and zoospores **(D)** in the control (circle), low N:P (triangle) and high N:P (square) treatments. Symbols represent mean ± standard error (*n* = 4). In **(C,D)** black, gray, and white symbols indicate carbon, nitrogen and phosphorus content, respectively. Solid lines indicate significant correlations (*P* < 0.05).

Host N:P ratios followed N:P supply (*r* = 0.99, *P* < 0.001; **Table 2**), and were lowest with 7.8 ± 0.1 (mean ± SE) under low N:P conditions, intermediate with 11.4 ± 0.4 in the control, and highest with 46.2 ± 2.7 under high N:P conditions (**Figure 2A**). This was also largely resembled in the overall N:P ratios of the cultures when the parasite was present (i.e., infected host + chytrids). The N:P ratio in the high N:P treatments was also highest with 44.2 ± 1.7, while the low N:P treatment and the control were not statistically different with an N:P ratio of 8.6 ± 0.22 and 10.3 ± 0.14, respectively. Similarly, N:P ratios of the chytrid zoospores increased with host N:P ratios (*r* = 0.96, *P* < 0.001). Specifically, chytrid N:P ratios increased from 12.6 ± 0.4 in the control to 25.9 ± 1.0 under high N:P, while remained largely unaltered in the low N:P treatment as compared to the control (**Figure 2B** and **Table 3**).

**Table 2:** Host nutrient content and stoichiometry (mean ± SE) in unexposed cultures.

	Nutrient content (10^-6^ μmol mm^-3^)			Stoichiometry (molar)		
Treatment	C	N	P	C:P	C:N	N:P
Low N:P	5148 ± 268^a^	837 ± 22^ab^	108 ± 3^a^	47.7 ± 1.9^a^	6.1 ± 0.2^a^	7.8 ± 0.1^a^
Control	4098 ± 120^b^	935 ± 26^a^	83 ± 4^b^	49.9 ± 1.9^a^	4.4 ± 0.0^b^	11.4 ± 0.4^b^
High N:P	3705 ± 242^b^	791 ± 48^b^	17 ± 1^c^	216.4 ± 11.7^b^	4.7 ± 0.1^b^	46.2 ± 2.7^c^

*Superscript letters denote significant differences between nutrient treatments based on One-way ANOVA and post hoc comparison of the means (α < 0.05).*

**Table 3:** Zoospore nutrient content and stoichiometry (mean ± SE).

	Nutrient content (×10^-4^ μmol per spore)			Stoichiometry (molar)		
Treatment	C	N	P	C:P	C:N	N:P
Low N:P	10.71 ± 0.77^a^	2.41 ± 0.18^a^	0.19 ± 0.01^a^	57.8 ± 0.7^a^	4.4 ± 0.0^a^	13.0 ± 0.2^a^
Control	5.27 ± 0.55^b^	1.11 ± 0.11^b^	0.09 ± 0.01^b^	59.9 ± 1.7^a^	4.7 ± 0.0^b^	12.6 ± 0.4^a^
High N:P	2.63 ± 0.49^c^	0.45 ± 0.08^c^	0.02 ± 0.00^c^	149.9 ± 6.5^b^	5.8 ± 0.1^c^	25.9 ± 1.0^b^

*Superscript letters denote significant differences between nutrient treatments based on One-way ANOVA and post hoc comparison of the means (α < 0.05).*

The observed shifts in host N:P ratios were mainly caused by changes in P contents (*r* = -0.97, *P* < 0.001), which decreased from 107.8 ± 2.5 pmol mm^-3^ at low N:P conditions down to 82.7 ± 4.3 pmol mm^-3^ in the control and 17.2 ± 1.0 pmol mm^-3^ under high N:P conditions, while C and N contents remained largely unaltered across all treatments (**Figure 2C** and **Table 2**). Zoospore C, N as well as P contents decreased with increasing host N:P (*r* = -0.77, *P* = 0.003, *r* = -0.77, *P* = 0.003 and *r* = -0.84, *P* < 0.001, respectively), with highest values under low N:P conditions, intermediate values in the control, and lowest values under high N:P conditions (**Figure 2D** and **Table 3**). The observed difference in N:P stoichiometry between high N:P conditions and the other nutrient supply treatments resulted from a stronger decline in P contents relative to N.

### Parasite Prevalence and Production

**FIGURE 3:**
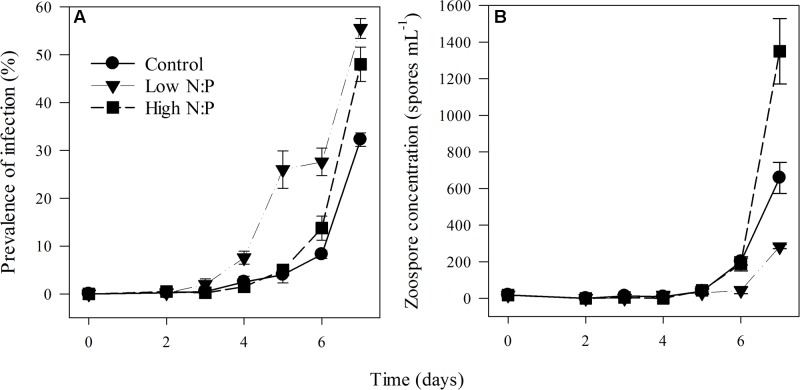
Prevalence of infection **(A)** and zoospore concentration **(B)** in the control, low N:P and high N:P treatments. Symbols represent mean ± standard error (*n* = 4).

**FIGURE 4:**
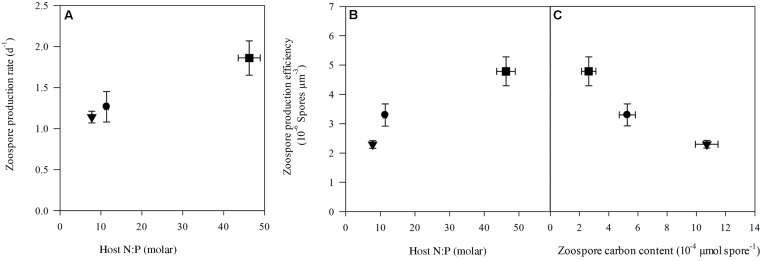
Zoospore production rate **(A)** and production efficiency **(B)** plotted against host N:P and zoospore carbon content **(C)** in the control (circle), low N:P (triangle) and high N:P (square) treatments. Symbols represent mean ± standard error (*n* = 4).

Prevalence of infection on the last day of the experiment ranged between 30 ± 1.4% (mean ± SE) in the control up to 48 ± 2.1% and 56 ± 3.6% in high N:P and low N:P treatments, respectively (**Figure 3A**). Growth rates of the infection were highest in the high N:P cultures and lowest, although not significantly, in the low N:P cultures (**Table 1**). Comparably, zoospore concentrations, zoospore production rate and the amount of zoospores produced per unit of infected host biomass, i.e., the zoospore production efficiency, were all highest in the high N:P cultures and lowest in low N:P cultures (**Figures 3B**, **4A,B** and **Table 4**). Zoospore production rate and efficiency increased with host N:P ratio (*r* = 0.61, *P* = 0.035, and *r* = 0.85, *P* < 0.001, respectively), while production efficiency furthermore decreased with zoospore C contents (*r* = -0.87, *P* < 0.001; **Figure 4C**).

**Table 4:** Zoospore production rates and production efficiencies (mean ± SE).

Treatment	Rate (d^-1^)	Efficiency (10^-6^ spores μm^-3^)
Low N:P	1.14 ± 0.07^a^	2.17 ± 0.04^a^
Control	1.27 ± 0.18^ab^	3.14 ± 0.42^b^
High N:P	1.86 ± 0.21^b^	5.02 ± 0.53^c^

*Superscript letters denote significant differences between nutrient treatments based on One-way ANOVA and post hoc comparison of the means (α < 0.05).*

### Microcystin

**FIGURE 5:**
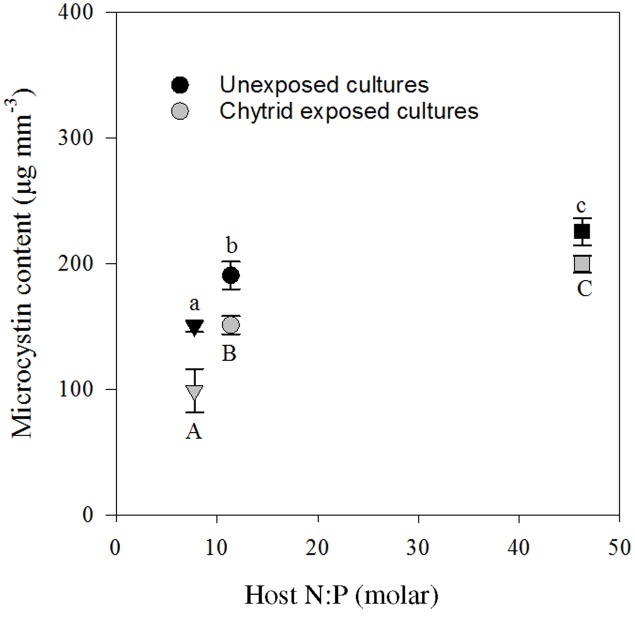
Seston microcystin content in the cultures with and without parasite exposure, in the control (circle), low N:P (triangle) and high N:P (square) treatments. Symbols represent mean ± standard error (*n* = 4). Letters denote significant differences between treatments of unexposed (lower case) and chytrid exposed (upper case) cultures based on One-way ANOVA and *post hoc* comparison of the means (α < 0.05).

Four MC variants were detected, including dm-7-MC-RR, MC-YR, dm-7-MC-LR, and MC-LR. On average, dm-7-MC-RR was the dominant MC variant present, representing 56.6 ± 0.5% (mean ± SE) of the total amount of MC. The total cellular MC contents ranged between 60 and 250 μg mm^-3^. MC concentrations were highest in the high N:P, lowest in the low N:P, and intermediate in the control treatment (**Figure 5**). Furthermore, in the chytrid exposed cultures, the total amount of intracellular MC seemed to be lower.

## Discussion

Although the different nutrient supply ratios had only minor effects on *Planktothrix* growth rates after acclimatization (**Table 1**), there were clear changes in the elemental composition of the cyanobacteria (**Figure 2A**). This indicates that nutrient depletion did affect host physiology, but not growth, at the time of sampling. Apparently, *Planktothrix* is able to maintain similar maximum growth rates as compared to the control at both a high and low N:P supply ratio (**Table 1**). The reduced host N:P under low N:P supply and increased host N:P under high N:P supply (**Figure 2A**) indicates nutrient limitation at the end of the exponential phase and/or early stationary phase. Moreover, population densities in the low N:P treatment at the end of the experiment were lower as compared to the control and high N:P treatment (**Figure 1B**). These lower population densities are mainly caused by a low N availability in the low N:P treatment, but may also result from the lower *Planktothrix* population densities at the start of the experiment. Differences in host population density may affect light availability in the cultures. A lowered population density, as observed in the low N:P treatment, may have resulted in an increased light availability due to reduced self-shading. Earlier studies have indicated that zoospores may find their host using chemical cues that are related to photosynthetic activity, since zoospores are generally attracted to carbohydrates, polysaccharides, proteins and amino acids (Muehlstein et al., 1988; Donaldson and Deacon, 1993; Moss et al., 2008). Some studies, however, also reported attachment of zoospores to new hosts during dark conditions (Barr and Hickman, 1967). Indirectly, the relative higher light availability in the low N:P treatment may thus have favored parasite attraction. Moreover, with a comparable amount of zoospores added at the start of the experiment, the zoospore-to-host ratio was also higher in the N limited treatment, which may favor infection rates. Yet, both the zoospore production rate and production efficiency were lower in the low N:P treatment, and did not lead to a different infection rate as compared to the control (**Table 1**). This suggests that higher relative light availabilities as well as higher initial zoospore-to-host ratios did not stimulate, and possibly even impeded the infection dynamics in our low N:P treatment.

In response to an increased N:P supply in the medium, host N:P increased as well. This resulted in a consecutive increase in the N:P of the zoospores (**Figure 2B**). Our results thus show that stoichiometry of a host can cascade to their parasites. In the host, changes in stoichiometry seem to be driven mainly by a change in P content, as host C and N remain constant with changing N:P supply (**Figure 2C**). In the parasite, changes in zoospore stoichiometry are also mainly driven by P content, but C and N contents decrease as well. Yet, P content decreases faster, suggesting a higher flexibility of the chytrid with respect to P (**Figure 2D**). These findings indicate that chytrid parasites can be stoichiometrically flexible, while maintaining their ability to infect along an N:P supply gradient. N:P and C:P ratios of the chytrid used in this experiment are relatively high as compared to two other studies using different chytrid species (Kagami et al., 2007, 2017), but fall well within the range of aquatic fungi reported before (Danger and Chauvet, 2013; Danger et al., 2015). These data furthermore suggest that fungal elemental homeostasis is indeed limited (Persson et al., 2010; Danger et al., 2015). Zoospore N:P ratios largely resembled that of the host under control conditions, but were different from the host in the low and high N:P treatment. We could not separate zoospores from the heterotrophic bacteria, and our results may therefore have been confounded by shifts in bacterial numbers. To prevent high bacterial numbers fueled by the lyses of *Planktothrix*, we ran the experiments over a relatively short time period. Consequently, the overall biomass of bacteria and thereby their contribution to the elemental composition at the time of sampling is likely to be small.

Increasing N:P supply ratios resulted in an increased zoospore production rate and production efficiency (**Figures 4A,B**). These results are in contrast to earlier findings of an experiment that showed that under P-limitation (and presumably high N:P) zoospore production decreased (Bruning, 1991). It might be possible that the chytrid species (*R. planktonicum* Canter emend.) used in the experiment by Bruning (1991) has higher P-requirements, and therefore suffered more from P-limitation. Or, because the chytrid in our experiment can potentially infect and exploit multiple adjacent cells within one cyanobacterial filament (Canter and Lund, 1951), it might be less vulnerable to nutrient limitation. In other words, the chytrid might continue infecting adjacent cells until it has consumed sufficient nutrients to complete an infection cycle. However, this is only profitable if the energetic costs of growing rhizoids and producing degrading enzymes to invade host cells balance the gains with respect to resource acquisition.

Under low N:P conditions, the parasite zoospores were fewer but contained more C, as well as N and P as compared to the control (**Figure 2D**). Although we did not assess zoospore size in this experiment directly, increases in elemental contents do suggest that the chytrid produced larger zoospores. Zoospore size of the used chytrid was shown to vary from 2.84 to 5.36 μm under control growth conditions (Supplementary Material), and variation in spore size was also shown in other studies describing shifts in spore size with climatic conditions (Kauserud et al., 2008, 2011). Presumably, larger zoospores facilitate zoospore survival time, since they can contain more lipids and fatty acids that might represent an energy source to fuel zoospore metabolism (Steinhoff et al., 2011). A longer spore survival time may be particularly favorable at lower host densities, and may explain the unaltered chytrid infections in the low N:P treatment. Conversely, in the high N:P conditions, more zoospores were produced per host biomass but contained less C, N and P per zoospore, suggesting that they were smaller. These findings are supported by earlier observations indicating that the efficiency of spore production by a parasitic dinoflagellate is increased under high N:P conditions, which might result in a higher transmission to new hosts under high host density conditions (Yih and Coats, 2000).

At low N:P supply the chytrid seems to produce a low amount of large zoospores, while at high N:P supply it produced a higher amount of small zoospores. The chytrid thus possibly produced smaller spores with a higher production efficiency (**Figure 4C**), suggesting a trade-off between size and production rate as well as success of infection. In other words, larger spores may survive longer providing the chytrid more time to find a suitable host under low host density conditions, while smaller ones survive less long but due to their high numbers achieve a higher infection transmission in high host density conditions. A trade-off between organism size and growth rate has also been reported for various other organisms, including phytoplankton (Nielsen, 2006) and zooplankton (Stemberger and Gilbert, 1985). Moreover, a trade-off between zoospore survival time and production rate was observed in another chytrid, the amphibian killing fungus *Batrachochytrium dendrobatidis* (Woodhams et al., 2008). Thus, changes in host N:P stoichiometry may affect the growth strategy of the parasite, following a more general trade-off between cell size and production rates (**Figure 4C**). Such changes can have consequences not only for the infection dynamics, but also for higher trophic levels that are provided with either many smaller zoospores, or fewer larger ones.

As expected, intracellular MC content closely followed the relative availability of N, and thus increased with cellular N:P ratios (**Figure 5**). These results are in line with earlier work, showing a strong dependency of MC contents on N availability (Van de Waal et al., 2009, 2010). In the treatment with high N:P supply and high MC production, however, zoospore production (**Figure 4A**) and infection rate (**Table 1**) were highest. Additionally, in the low N:P treatment, MC contents was lowest while zoospore production and infection rate were not different from the control. So, there was no clear relation between intracellular MC content and chytrid proliferation. Possibly, a considerable fraction of the total MC might be bound to proteins of cyanobacterial cells (Zilliges et al., 2011), which were not included in our extraction processes. Furthermore, *Planktothrix* may produce other oligopeptides that play a role in parasite defense systems (Sønstebø and Rohrlack, 2011; Rohrlack et al., 2013), which were not analyzed here.

The intracellular MC seemed to be lower in the parasite exposed treatments as compared to the unexposed treatments. This may possibly result from leakage of MC from the cells into the liquid phase (Jones and Orr, 1994). Moreover, chytrid rhizoids that invade the host cells might use enzymes that are able to digest MC. Indeed, fungi were shown to be capable of degrading MC (Jia et al., 2012). In our experiment, however, extracellular MC concentrations nor chytrid MC contents were analyzed. If MC is released into the water column from cyanobacterial cells, it can have consequences for other organisms present (Carmichael, 1992; Zurawell et al., 2005). For instance, high MC concentrations in the water can accumulate in *Daphnia* (Chen et al., 2005) and have adverse effects on growth and development of fish (Jacquet et al., 2004). Yet, actual exposure of other organisms to MC in the water may be limited, as MCs can be rapidly biodegraded and detoxified by bacteria and adsorb to plants and sediments (Harada and Tsuji, 1998; Pflugmacher et al., 2001; Kato et al., 2007). Whether MCs can bind to- or be transported into zoospores is unknown. But, if this would occur, zooplankton might be exposed to MCs via this indirect route, since zoospores can serve as a food source for copepods, cladocerans and possibly rotifers (Kagami et al., 2004, 2007, 2011; Buck et al., 2011; Agha et al., 2016; Frenken et al., 2016).

Our results demonstrate an increase in infection rate with host N:P stoichiometry, thereby showing the opposite to what we hypothesized. Because chytrids seemed relatively more P rich as compared to their host, we initially predicted that host P content would constrain chytrid growth more than it would constrain the host (Bruning and Ringelberg, 1987; Bruning, 1991). Our results suggest, however, that chytrid proliferation is much more sensitive to the relative availability of N. Specifically, if this increases (i.e., higher host N:P), infection rates increase, while if this decreases (i.e., lower host N:P), infection rates decrease. This is also shown by the lower flexibility of the parasite N content as compared to P, suggesting that spores are more likely to be constrained under low N conditions. It remains unclear why infection rates increase under P limitation and relative high N contents. Particularly as under these high N:P conditions, MC contents were highest as well. We initially expected that under such conditions, chytrid infections can be inhibited by MCs in its host. The increase in MC content with high N:P conditions, however, was relatively small and may therefore not have been sufficient to inhibit the chytrid infection. Possibly, regulation of other oligopeptides in response to N:P supply could have explained the observed responses, and should thus be included in future analyzes. Moreover, other metabolites synthesized by cyanobacteria under high N:P supply may have facilitated chytrid growth and reproduction. Further detailed biochemical analyses of chytrids and their distinct developmental stages would be required to fully understand the stoichiometric interactions with their hosts, and particularly the putative important role of N in controlling infections.

Our analysis revealed some still poorly understood effects of nutrient availability on the interaction of a host–parasite system. Shifts in nutrient supply ratios not only lead to a shift in host stoichiometry, but also to comparable changes in the parasite. Thereby, we show that elemental stoichiometry of a host can cascade to their parasites. We hypothesize that, in response to changes in nutrient supply, the parasite may exhibit a trade-off between size and zoospore production rate to optimize reproductive success. Therefore, nutrient limitation may indirectly affect parasite abundance and stoichiometry. Since chytrids can facilitate growth of zooplankton (Kagami et al., 2007; Agha et al., 2016), changes in parasite production, stoichiometry and cell size may have implications for aquatic food web dynamics.

## Author Contributions

TF, JW, and DVdW designed the study. JW and TF performed the experiment. TF, JW, AG, and DVdW analyzed and interpreted the data. TF and DVdW wrote a first draft of the manuscript which was corrected, revised and approved by all authors.

## Conflict of Interest Statement

The authors declare that the research was conducted in the absence of any commercial or financial relationships that could be construed as a potential conflict of interest.
