# Supplementary material for: Changes in N:P Supply Ratios Affect the Ecological Stoichiometry of a Toxic Cyanobacterium and Its Fungal Parasite
Source: Front Microbiol. 2017 Jun 6;8:1015. doi: 10.3389/fmicb.2017.01015 (PMC5459933; doi:10.3389/fmicb.2017.01015)
Supplement: Supplementary file 1 [file Data_Sheet_1.DOCX]

Supplementary Material

**Changes in N:P Supply Ratios affect the Ecological Stoichiometry of a Toxic Cyanobacterium and its Fungal Parasite**

**Thijs Frenken^*^, Joren Wierenga, Alena S. Gsell, Ellen Van Donk ,Thomas Rohrlack, Dedmer B. Van de Waal**

*** Correspondence:** Corresponding Author: [T.Frenken@nioo.knaw.nl](mailto:T.Frenken@nioo.knaw.nl)

# Supplementary Figures

*Picture of* the filamentous cyanobacterial *Planktothrix rubescens* NIVA-CYA97/1 with its parasite ,the chytrid Chy-Lys2009. (A) Healthy filament, (B) Young infection, (C) Mature infection (scale bar length equals 50 µm in A+B and 20 µm in C).


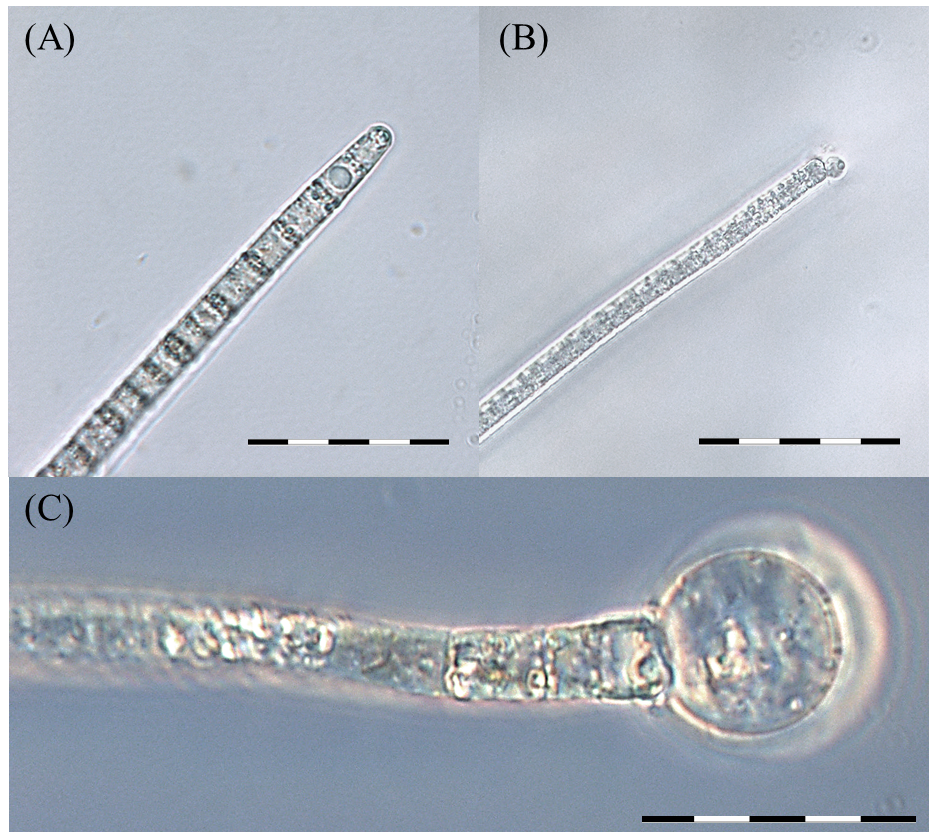


*Zoospore size measurements*

Method:

Zoospore sizes were checked in two different infected *Planktothrix* cultures grown under equal nutrient conditions as the control treatments from this experiment. Samples were fixed to a final concentration with 0.5% (v/v) with 25% glutaraldehyde (Merck, Darmstadt, Germany). Samples were left to settle overnight. Then, in each sample, 75 zoospore diameters were measured on an inverted microscope (DMI 4000B; Leica Microsystems CMS GmbH, Mannheim, Germany) using the software of Soft Imaging System Cell D version 1.20 (Soft Imaging System GmbH, Münster, Germany).

Result:

Zoospore diameter can vary from 2.84 µm up to 5.36 µm. On average, zoospore diameter is 4.02±0.48 um (Mean±StDev) and 4.11±0.58 for sample 1 and sample 2 respectively (see also Fig S1).


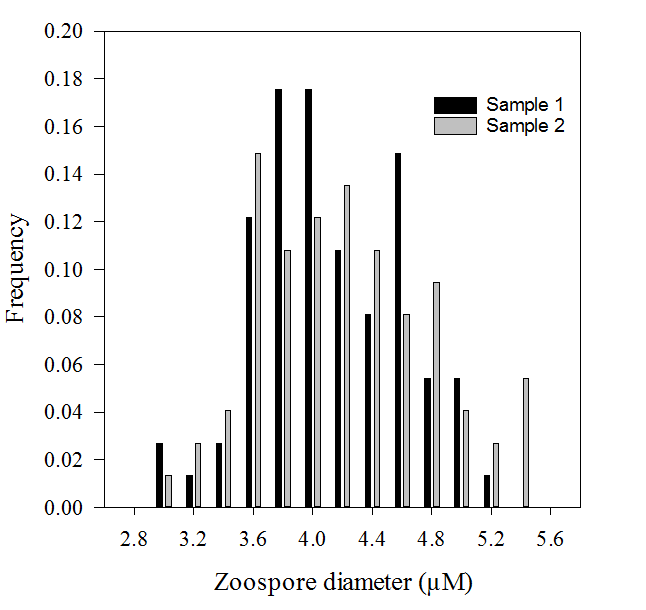


Figure S1: Histogram of zoospore diameter measured in two different infected *Planktothrix* cultures grown under nutrient rich conditions resembling the control treatment from this experiment.

# Supplementary Tables

*Output One Way Analysis of Variance*

| Reciprocal host growth rate unexposed | |  |  |  |  |
| --- | --- | --- | --- | --- | --- |
| **One Way Analysis of Variance** |  |  |  |  |  |
| Group Name | N | Missing | Mean | Std Dev | SEM |
| control | 4 | 0 | 2.577 | 0.515 | 0.257 |
| Low N:P | 4 | 0 | 2.509 | 0.522 | 0.261 |
| High N:P | 4 | 0 | 2.653 | 0.684 | 0.342 |
|  |  |  |  |  |  |
| Source of Variation | DF | SS | MS | F | P |
| Between Groups | 2 | 0.0419 | 0.021 | 0.0626 | 0.94 |
| Residual | 9 | 3.014 | 0.335 |  |  |
| Total | 11 | 3.056 |  |  |  |

| Host growth rate exposed |  |  |  |  |  |
| --- | --- | --- | --- | --- | --- |
| **One Way Analysis of Variance** |  |  |  |  |  |
| Group Name | N | Missing | Mean | Std Dev | SEM |
| control | 4 | 0 | 0.113 | 0.0745 | 0.0373 |
| Low N:P | 4 | 0 | 0.0611 | 0.0923 | 0.0461 |
| High N:P | 4 | 0 | 0.112 | 0.0646 | 0.0323 |
|  |  |  |  |  |  |
| Source of Variation | DF | SS | MS | F | P |
| Between Groups | 2 | 0.00707 | 0.00354 | 0.582 | 0.579 |
| Residual | 9 | 0.0547 | 0.00608 |  |  |
| Total | 11 | 0.0618 |  |  |  |

| Growth rate susceptible biomass |  |  |  |  |  |
| --- | --- | --- | --- | --- | --- |
| **One Way Analysis of Variance** |  |  |  |  |  |
| Group Name | N | Missing | Mean | Std Dev | SEM |
| control | 4 | 0 | 0.0993 | 0.0611 | 0.0306 |
| Low N:P | 4 | 0 | -0.0459 | 0.0447 | 0.0224 |
| High N:P | 4 | 0 | 0.0965 | 0.0699 | 0.0349 |
|  |  |  |  |  |  |
| Source of Variation | DF | SS | MS | F | P |
| Between Groups | 2 | 0.0552 | 0.0276 | 7.795 | 0.011 |
| Residual | 9 | 0.0319 | 0.00354 |  |  |
| Total | 11 | 0.087 |  |  |  |
|  |  |  |  |  |  |
| Comparisons for factor: Nutrient |  |  |  |  |  |
| Comparison | Diff of Means | t | P | P<0.050 |  |
| High N:P vs. Low N:P | 0.142 | 3.385 | 0.016 | Yes |  |
| High N:P vs. control | 0.00282 | 0.067 | 0.948 | No |  |
| control vs. Low N:P | 0.145 | 3.452 | 0.022 | Yes |  |

| Growth rate infected biomass |  |  |  |  |  |
| --- | --- | --- | --- | --- | --- |
| **One Way Analysis of Variance** |  |  |  |  |  |
| Group Name | N | Missing | Mean | Std Dev | SEM |
| control | 4 | 0 | 0.919 | 0.19 | 0.0949 |
| Low N:P | 4 | 0 | 0.944 | 0.171 | 0.0854 |
| High N:P | 4 | 0 | 1.337 | 0.177 | 0.0886 |
|  |  |  |  |  |  |
| Source of Variation | DF | SS | MS | F | P |
| Between Groups | 2 | 0.44 | 0.22 | 6.825 | 0.016 |
| Residual | 9 | 0.29 | 0.0322 |  |  |
| Total | 11 | 0.73 |  |  |  |
|  |  |  |  |  |  |
| Comparisons for factor: Nutrient |  |  |  |  |  |
| Comparison | Diff of Means | t | P | P<0.050 |  |
| High N:P vs. Low N:P | 0.393 | 3.1 | 0.025 | Yes |  |
| High N:P vs. control | 0.418 | 3.291 | 0.028 | Yes |  |
| control vs. Low N:P | 0.0243 | 0.191 | 0.853 | No |  |

| Log host N:P |  |  |  |  |  |
| --- | --- | --- | --- | --- | --- |
| **One Way Analysis of Variance** |  |  |  |  |  |
| Group Name | N | Missing | Mean | Std Dev | SEM |
| control | 4 | 0 | 1.055 | 0.0334 | 0.0167 |
| Low N:P | 4 | 0 | 0.89 | 0.0132 | 0.00658 |
| High N:P | 4 | 0 | 1.663 | 0.0485 | 0.0242 |
|  |  |  |  |  |  |
| Source of Variation | DF | SS | MS | F | P |
| Between Groups | 2 | 1.326 | 0.663 | 546.838 | <0.001 |
| Residual | 9 | 0.0109 | 0.00121 |  |  |
| Total | 11 | 1.337 |  |  |  |
|  |  |  |  |  |  |
| Comparisons for factor: Nutrient | |  |  |  |  |
| Comparison | Diff of Means | t | P | P<0.050 |  |
| High N:P vs. Low N:P | 0.773 | 31.402 | <0.001 | Yes |  |
| High N:P vs. control | 0.608 | 24.683 | <0.001 | Yes |  |
| control vs. Low N:P | 0.165 | 6.719 | <0.001 | Yes |  |

| Log zoospore N:P | | |  |  |  |
| --- | --- | --- | --- | --- | --- |
| **One Way Analysis of Variance** | | |  |  |  |
| Group Name | N | Missing | Mean | Std Dev | SEM |
| control | 4 | 0 | 1.101 | 0.0248 | 0.0124 |
| Low N:P | 4 | 0 | 1.114 | 0.0127 | 0.00636 |
| High N:P | 4 | 0 | 1.412 | 0.0317 | 0.0159 |
|  |  |  |  |  |  |
| Source of Variation | DF | SS | MS | F | P |
| Between Groups | 2 | 0.249 | 0.124 | 209.368 | <0.001 |
| Residual | 9 | 0.00535 | 0.000594 |  |  |
| Total | 11 | 0.254 |  |  |  |
|  |  |  |  |  |  |
| Comparisons for factor: Nutrient | | | |  |  |
| Comparison | Diff of Means | t | P | P<0.050 |  |
| High N:P vs. Low N:P | 0.312 | 18.082 | <0.001 | Yes |  |
| High N:P vs. control | 0.299 | 17.337 | <0.001 | Yes |  |
| control vs. Low N:P | 0.0128 | 0.745 | 0.475 | No |  |

| Zoospore production rate |  |  |  |  |  |
| --- | --- | --- | --- | --- | --- |
| **One Way Analysis of Variance** |  |  |  |  |  |
| Group Name | N | Missing | Mean | Std Dev | SEM |
| control | 4 | 0.00 | 1.27 | 0.369 | 0.185 |
| Low N:P | 4 | 0.00 | 1.14 | 0.144 | 0.0722 |
| High N:P | 4 | 0.00 | 1.86 | 0.42 | 0.21 |
|  |  |  |  |  |  |
| Source of Variation | DF | SS | MS | F | P |
| Between Groups | 2 | 1.18 | 0.59 | 5.312 | 0.03 |
| Residual | 9 | 1 | 0.111 |  |  |
| Total | 11 | 2.18 |  |  |  |
|  |  |  |  |  |  |
| Comparisons for factor: Nutrient | |  |  |  |  |
| Comparison | Diff of Means | t | P | P<0.050 |  |
| High N:P vs. Low N:P | 0.719 | 3.051 | 0.041 | Yes |  |
| High N:P vs. control | 0.593 | 2.518 | 0.065 | No |  |
| control vs. Low N:P | 0.126 | 0.533 | 0.607 | No |  |

| Log zoospore production efficiency | |  |  |  |  |
| --- | --- | --- | --- | --- | --- |
| **One Way Analysis of Variance** |  |  |  |  |  |
| Group Name | N | Missing | Mean | Std Dev | SEM |
| control | 4 | 0.00 | -5.49 | 0.109 | 0.0543 |
| Low N:P | 4 | 0.00 | -5.64 | 0.0476 | 0.0238 |
| High N:P | 4 | 0.00 | -5.33 | 0.0881 | 0.0441 |
|  |  |  |  |  |  |
| Source of Variation | DF | SS | MS | F | P |
| Between Groups | 2 | 0.198 | 0.099 | 13.627 | 0.002 |
| Residual | 9 | 0.0654 | 0.00727 |  |  |
| Total | 11 | 0.263 |  |  |  |
|  |  |  |  |  |  |
| Comparisons for factor: Nutrient | |  |  |  |  |
| Comparison | Diff of Means | t | P | P<0.050 |  |
| High N:P vs. Low N:P | 0.315 | 5.219 | 0.002 | Yes |  |
| High N:P vs. control | 0.165 | 2.733 | 0.046 | Yes |  |
| control vs. Low N:P | 0.15 | 2.486 | 0.035 | Yes |  |

| Log host C:P | |  |  |  |  |
| --- | --- | --- | --- | --- | --- |
| **One Way Analysis of Variance** | | | |  |  |
| Group Name | N | Missing | Mean | Std Dev | SEM |
| control | 4 | 0 | 1.697 | 0.033 | 0.0165 |
| Low N:P | 4 | 0 | 1.678 | 0.034 | 0.017 |
| High N:P | 4 | 0 | 2.333 | 0.0456 | 0.0228 |
|  |  |  |  |  |  |
| Source of Variation | DF | SS | MS | F | P |
| Between Groups | 2 | 1.11E+00 | 5.57E-01 | 385.986 | <0.001 |
| Residual | 9 | 1.30E-02 | 1.44E-03 |  |  |
| Total | 11 | 1.13E+00 |  |  |  |
|  |  |  |  |  |  |
| Comparisons for factor: Nutrient | |  |  |  |  |
| Comparison | Diff of Means | t | P | P<0.050 |  |
| high N:P vs. low N:P | 0.656 | 3 | 34.532 | <0.001 | Yes |
| high N:P vs. control | 0.636 | 3 | 33.502 | <0.001 | Yes |
| control vs. low N:P | 0.0196 | 3 | 1.031 | 0.753 | No |

| Log host C:N | |  |  |  |  |
| --- | --- | --- | --- | --- | --- |
| **One Way Analysis of Variance** | | | |  |  |
| Group Name | N | Missing | Mean | Std Dev | SEM |
| control | 4 | 0 | 0.641 | 0.00187 | 0.000933 |
| Low N:P | 4 | 0 | 0.788 | 0.0276 | 0.0138 |
| High N:P | 4 | 0 | 0.67 | 0.00937 | 0.00468 |
|  |  |  |  |  |  |
| Source of Variation | DF | SS | MS | F | P |
| Between Groups | 2 | 0.0483 | 0.0241 | 85.092 | <0.001 |
| Residual | 9 | 0.00255 | 0.000284 |  |  |
| Total | 11 | 0.0508 |  |  |  |
|  |  |  |  |  |  |
| Comparisons for factor: Nutrient | |  |  |  |  |
| Comparison | Diff of Means | t | P | P<0.050 |  |
| low N:P vs. control | 0.147 | 3 | 17.412 | <0.001 | Yes |
| low N:P vs. high N:P | 0.118 | 3 | 13.988 | <0.001 | Yes |
| high N:P vs. control | 0.0288 | 3 | 3.423 | 0.089 | No |

| Host C | |  |  |  |  |
| --- | --- | --- | --- | --- | --- |
| **One Way Analysis of Variance** | | | |  |  |
| Group Name | N | Missing | Mean | Std Dev | SEM |
| control | 4 | 0 | 4098.49 | 239.227 | 119.613 |
| Low N:P | 4 | 0 | 5147.796 | 535.566 | 267.783 |
| High N:P | 4 | 0 | 3704.938 | 484.431 | 242.215 |
|  |  |  |  |  |  |
| Source of Variation | DF | SS | MS | F | P |
| Between Groups | 2 | 4450358 | 2225179 | 11.535 | 0.003 |
| Residual | 9 | 1736202 | 192911.4 |  |  |
| Total | 11 | 6186560 |  |  |  |
|  |  |  |  |  |  |
| Comparisons for factor: Nutrient | |  |  |  |  |
| Comparison | Diff of Means | t | P | P<0.050 |  |
| low N:P vs. high N:P | 1442.859 | 3 | 6.57 | 0.003 | Yes |
| low N:P vs. control | 1049.307 | 3 | 4.778 | 0.02 | Yes |
| control vs. high N:P | 393.552 | 3 | 1.792 | 0.447 | No |

| Host N | |  |  |  |  |
| --- | --- | --- | --- | --- | --- |
| **One Way Analysis of Variance** | | | |  |  |
| Group Name | N | Missing | Mean | Std Dev | SEM |
| control | 4 | 0 | 934.724 | 51.512 | 25.756 |
| Low N:P | 4 | 0 | 836.675 | 44.251 | 22.126 |
| High N:P | 4 | 0 | 790.704 | 95.956 | 47.978 |
|  |  |  |  |  |  |
| Source of Variation | DF | SS | MS | F | P |
| Between Groups | 2 | 43291.36 | 21645.68 | 4.699 | 0.04 |
| Residual | 9 | 41457.67 | 4606.408 |  |  |
| Total | 11 | 84749.03 |  |  |  |
|  |  |  |  |  |  |
| Comparisons for factor: Nutrient | |  |  |  |  |
| Comparison | Diff of Means | t | P | P<0.050 |  |
| control vs. high N:P | 144.02 | 3 | 4.244 | 0.036 | Yes |
| control vs. low N:P | 98.049 | 3 | 2.889 | 0.158 | No |
| low N:P vs. high N:P | 45.971 | 3 | 1.355 | 0.62 | No |

| Host P | |  |  |  |  |
| --- | --- | --- | --- | --- | --- |
| **One Way Analysis of Variance** | | | |  |  |
| Group Name | N | Missing | Mean | Std Dev | SEM |
| control | 4 | 0 | 82.697 | 8.512 | 4.256 |
| Low N:P | 4 | 0 | 107.782 | 5.064 | 2.532 |
| High N:P | 4 | 0 | 17.195 | 1.966 | 0.983 |
|  |  |  |  |  |  |
| Source of Variation | DF | SS | MS | F | P |
| Between Groups | 2 | 17501 | 8750.5 | 257.439 | <0.001 |
| Residual | 9 | 305.915 | 33.991 |  |  |
| Total | 11 | 17806.92 |  |  |  |
|  |  |  |  |  |  |
| Comparisons for factor: Nutrient | |  |  |  |  |
| Comparison | Diff of Means | t | P | P<0.050 |  |
| low N:P vs. high N:P | 90.587 | 3 | 31.075 | <0.001 | Yes |
| low N:P vs. control | 25.084 | 3 | 8.605 | <0.001 | Yes |
| control vs. high N:P | 65.503 | 3 | 22.47 | <0.001 | Yes |

| Zoospore C | |  |  |  |  |
| --- | --- | --- | --- | --- | --- |
| **One Way Analysis of Variance** | | | |  |  |
| Group Name | N | Missing | Mean | Std Dev | SEM |
| control | 4 | 0 | 5.267 | 1.098 | 0.549 |
| Low N:P | 4 | 0 | 10.712 | 1.547 | 0.774 |
| High N:P | 4 | 0 | 2.63 | 0.971 | 0.486 |
|  |  |  |  |  |  |
| Source of Variation | DF | SS | MS | F | P |
| Between Groups | 2 | 135.893 | 67.946 | 44.872 | <0.001 |
| Residual | 9 | 13.628 | 1.514 |  |  |
| Total | 11 | 149.521 |  |  |  |
|  |  |  |  |  |  |
| Comparisons for factor: Nutrient | |  |  |  |  |
| Comparison | Diff of Means | t | P | P<0.050 |  |
| low N:P vs. high N:P | 8.082 | 9.288 | <0.001 | Yes |  |
| low N:P vs. control | 5.445 | 6.257 | <0.001 | Yes |  |
| control vs. high N:P | 2.638 | 3.031 | 0.014 | Yes |  |

| Zoospore N | |  |  |  |  |
| --- | --- | --- | --- | --- | --- |
| **One Way Analysis of Variance** | | | |  |  |
| Group Name | N | Missing | Mean | Std Dev | SEM |
| control | 4 | 0 | 1.109 | 0.224 | 0.112 |
| Low N:P | 4 | 0 | 2.411 | 0.364 | 0.182 |
| High N:P | 4 | 0 | 0.454 | 0.166 | 0.0829 |
|  |  |  |  |  |  |
| Source of Variation | DF | SS | MS | F | P |
| Between Groups | 2 | 7.936 | 3.968 | 56.781 | <0.001 |
| Residual | 9 | 0.629 | 0.0699 |  |  |
| Total | 11 | 8.565 |  |  |  |
|  |  |  |  |  |  |
| Comparisons for factor: Nutrient | |  |  |  |  |
| Comparison | Diff of Means | t | P | P<0.050 |  |
| low N:P vs. high N:P | 1.957 | 10.467 | <0.001 | Yes |  |
| low N:P vs. control | 1.302 | 6.965 | <0.001 | Yes |  |
| control vs. high N:P | 0.655 | 3.502 | 0.007 | Yes |  |

| Zoospore P | |  |  |  |  |
| --- | --- | --- | --- | --- | --- |
| **One Way Analysis of Variance** | | | |  |  |
| Group Name | N | Missing | Mean | Std Dev | SEM |
| control | 4 | 0 | 0.0876 | 0.0161 | 0.00806 |
| Low N:P | 4 | 0 | 0.185 | 0.0225 | 0.0113 |
| High N:P | 4 | 0 | 0.0173 | 0.00497 | 0.00248 |
|  |  |  |  |  |  |
| Source of Variation | DF | SS | MS | F | P |
| Between Groups | 2 | 0.0568 | 0.0284 | 107.698 | <0.001 |
| Residual | 9 | 0.00237 | 0.000264 |  |  |
| Total | 11 | 0.0592 |  |  |  |
|  |  |  |  |  |  |
| Comparisons for factor: Nutrient | |  |  |  |  |
| Comparison | Diff of Means | t | P | P<0.050 |  |
| low N:P vs. high N:P | 0.168 | 14.613 | <0.001 | Yes |  |
| low N:P vs. control | 0.0975 | 8.49 | <0.001 | Yes |  |
| control vs. high N:P | 0.0703 | 6.123 | <0.001 | Yes |  |

| Zoospore C:P | |  |  |  |  |
| --- | --- | --- | --- | --- | --- |
| **One Way Analysis of Variance** | | | |  |  |
| Group Name | N | Missing | Mean | Std Dev | SEM |
| control | 4 | 0 | 59.914 | 3.496 | 1.748 |
| Low N:P | 4 | 0 | 57.759 | 1.33 | 0.665 |
| High N:P | 4 | 0 | 149.938 | 12.922 | 6.461 |
|  |  |  |  |  |  |
| Source of Variation | DF | SS | MS | F | P |
| Between Groups | 2 | 22141.26 | 11070.63 | 183.533 | <0.001 |
| Residual | 9 | 542.876 | 60.32 |  |  |
| Total | 11 | 22684.14 |  |  |  |
|  |  |  |  |  |  |
| Comparisons for factor: Nutrient | |  |  |  |  |
| Comparison | Diff of Means | t | P | P<0.050 |  |
| low N:P vs. high N:P | 92.179 | 16.785 | <0.001 | Yes |  |
| low N:P vs. control | 2.155 | 0.392 | 0.704 | No |  |
| control vs. high N:P | 90.024 | 16.392 | <0.001 | Yes |  |

| Zoospore C:N | |  |  |  |  |
| --- | --- | --- | --- | --- | --- |
| **One Way Analysis of Variance** | | | |  |  |
| Group Name | N | Missing | Mean | Std Dev | SEM |
| control | 4 | 0 | 12.625 | 0.714 | 0.357 |
| Low N:P | 4 | 0 | 12.993 | 0.384 | 0.192 |
| High N:P | 4 | 0 | 25.896 | 1.928 | 0.964 |
|  |  |  |  |  |  |
| Source of Variation | DF | SS | MS | F | P |
| Between Groups | 2 | 456.993 | 228.497 | 156.684 | <0.001 |
| Residual | 9 | 13.125 | 1.458 |  |  |
| Total | 11 | 470.118 |  |  |  |
|  |  |  |  |  |  |
| Comparisons for factor: Nutrient | |  |  |  |  |
| Comparison | Diff of Means | t | P | P<0.050 |  |
| low N:P vs. high N:P | 1.34 | 22.84 | <0.001 | Yes |  |
| low N:P vs. control | 0.299 | 5.097 | <0.001 | Yes |  |
| control vs. high N:P | 1.041 | 17.743 | <0.001 | Yes |  |

| host MC content unexposed |  |  |  |  |  |
| --- | --- | --- | --- | --- | --- |
| **One Way Analysis of Variance** |  |  |  |  |  |
| Group Name | N | Missing | Mean | Std Dev | SEM |
| control | 4 | 0 | 1.91E-07 | 2.21E-08 | 1.1E-08 |
| Low N:P | 4 | 0 | 1.5E-07 | 8.98E-09 | 4.49E-09 |
| High N:P | 4 | 0 | 2.26E-07 | 2.17E-08 | 1.08E-08 |
|  |  |  |  |  |  |
| Source of Variation | DF | SS | MS | F | P |
| Between Groups | 2 | 1.14E-14 | 5.71E-15 | 16.489 | <0.001 |
| Residual | 9 | 3.12E-15 | 3.46E-16 |  |  |
| Total | 11 | 1.45E-14 |  |  |  |
|  |  |  |  |  |  |
| Comparisons for factor: Nutrient |  |  |  |  |  |
| Comparison | Diff of Means | t | P | P<0.050 |  |
| High N:P vs. Low N:P | 7.55E-08 | 5.738 | <0.001 | Yes |  |
| High N:P vs. control | 3.5E-08 | 2.66 | 0.026 | Yes |  |
| control vs. Low N:P | 4.05E-08 | 3.078 | 0.026 | Yes |  |

| host MC content exposed |  |  |  |  |  |
| --- | --- | --- | --- | --- | --- |
| One Way Analysis of Variance |  |  |  |  |  |
| Group Name | N | Missing | Mean | Std Dev | SEM |
| control | 4 | 0 | 1.51E-07 | 1.45E-08 | 7.27E-09 |
| Low N:P | 4 | 0 | 9.91E-08 | 3.46E-08 | 1.73E-08 |
| High N:P | 4 | 0 | 2E-07 | 1.32E-08 | 6.6E-09 |
|  |  |  |  |  |  |
| Source of Variation | DF | SS | MS | F | P |
| Between Groups | 2 | 2.03E-14 | 1.01E-14 | 19.185 | <0.001 |
| Residual | 9 | 4.76E-15 | 5.29E-16 |  |  |
| Total | 11 | 2.5E-14 |  |  |  |
|  |  |  |  |  |  |
| Comparisons for factor: Nutrient |  |  |  |  |  |
| Comparison | Diff of Means | t | P | P<0.050 |  |
| High N:P vs. Low N:P | 1.01E-07 | 6.193 | <0.001 | Yes |  |
| High N:P vs. control | 4.86E-08 | 2.991 | 0.015 | Yes |  |
| control vs. Low N:P | 5.21E-08 | 3.202 | 0.021 | Yes |  |

*Output linear regression (Pearson product moment correlation)*

Host N:P vs. Treatment N:P

| Host N:P = 4.763 + (0.412 * Treatment N:P) | | | | |  |
| --- | --- | --- | --- | --- | --- |
|  |  |  |  |  |  |
| N = 12 |  |  |  |  |  |
|  |  |  |  |  |  |
| R = 0.986 | Rsqr = 0.971 | Adj Rsqr = 0.968 | |  |  |
|  |  |  |  |  |  |
| Standard Error of Estimate = 3.256 | | | |  |  |
|  |  |  |  |  |  |
|  | Coefficient | Std. Error | t | P |  |
| Constant | 4.763 | 1.319 | 3.612 | 0.005 |  |
| Treatment N:P | 0.412 | 0.0224 | 18.411 | <0.001 |  |
|  |  |  |  |  |  |
| Analysis of Variance: | | |  |  |  |
|  | DF | SS | MS | F | P |
| Regression | 1 | 3594.016 | 3594.016 | 338.952 | <0.001 |
| Residual | 10 | 106.033 | 10.603 |  |  |
| Total | 11 | 3700.049 | 336.368 |  |  |

Spore N:P vs. Host N:P

| Spore N:P = 9.735 + (0.341 * Host N:P) | | | |  |  |
| --- | --- | --- | --- | --- | --- |
|  |  |  |  |  |  |
| N = 12 |  |  |  |  |  |
|  |  |  |  |  |  |
| R = 0.957 | Rsqr = 0.916 | Adj Rsqr = 0.908 | |  |  |
|  |  |  |  |  |  |
| Standard Error of Estimate = 1.986 | | | |  |  |
|  |  |  |  |  |  |
|  | Coefficient | Std. Error | t | P |  |
| Constant | 9.735 | 0.914 | 10.653 | <0.001 |  |
| Host N:P | 0.341 | 0.0326 | 10.45 | <0.001 |  |
|  |  |  |  |  |  |
| Analysis of Variance: | | |  |  |  |
|  | DF | SS | MS | F | P |
| Regression | 1 | 430.68 | 430.68 | 109.204 | <0.001 |
| Residual | 10 | 39.438 | 3.944 |  |  |
| Total | 11 | 470.118 | 42.738 |  |  |

| Host c = 0.00000487 - (0.0000000254 * Host N:P) | | | | |  |
| --- | --- | --- | --- | --- | --- |
|  |  |  |  |  |  |
| N = 12 |  |  |  |  |  |
|  |  |  |  |  |  |
| R = 0.622 | Rsqr = 0.387 | Adj Rsqr = 0.326 | |  |  |
|  |  |  |  |  |  |
| Standard Error of Estimate = 0.000 | | | |  |  |
|  |  |  |  |  |  |
|  | Coefficient | Std. Error | t | P |  |
| Constant | 4.87E-06 | 2.83E-07 | 17.191 | <0.001 |  |
| Host N:P | -2.5E-08 | 1.01E-08 | -2.513 | 0.031 |  |
|  |  |  |  |  |  |
| Analysis of Variance: | | |  |  |  |
|  | DF | SS | MS | F | P |
| Regression | 1 | 2.39E-12 | 2.39E-12 | 6.313 | 0.031 |
| Residual | 10 | 3.79E-12 | 3.79E-13 |  |  |
| Total | 11 | 6.19E-12 | 5.62E-13 |  |  |

Host c vs. Host N:P

Host n vs. Host N:P

| Host N = 0.000000900 - (0.00000000210 * Host N:P) | | | | |  |
| --- | --- | --- | --- | --- | --- |
|  |  |  |  |  |  |
| N = 12 |  |  |  |  |  |
|  |  |  |  |  |  |
| R = 0.439 | Rsqr = 0.193 | Adj Rsqr = 0.112 | |  |  |
|  |  |  |  |  |  |
| Standard Error of Estimate = 0.000 | | | |  |  |
|  |  |  |  |  |  |
|  | Coefficient | Std. Error | t | P |  |
| Constant | 9E-07 | 3.81E-08 | 23.647 | <0.001 |  |
| Host N:P | -2.1E-09 | 1.36E-09 | -1.547 | 0.153 |  |
|  |  |  |  |  |  |
| Analysis of Variance: | | |  |  |  |
|  | DF | SS | MS | F | P |
| Regression | 1 | 1.64E-14 | 1.64E-14 | 2.392 | 0.153 |
| Residual | 10 | 6.84E-14 | 6.84E-15 |  |  |
| Total | 11 | 8.48E-14 | 7.70E-15 |  |  |

Host p vs. Host N:P

| Host P = 0.000000115 - (0.00000000212 * Host N:P) | | | | |  |
| --- | --- | --- | --- | --- | --- |
|  |  |  |  |  |  |
| N = 12 |  |  |  |  |  |
|  |  |  |  |  |  |
| R = 0.967 | Rsqr = 0.934 | Adj Rsqr = 0.928 | |  |  |
|  |  |  |  |  |  |
| Standard Error of Estimate = 0.000 | | | |  |  |
|  |  |  |  |  |  |
|  | Coefficient | Std. Error | t | P |  |
| Constant | 1.15E-07 | 4.98E-09 | 23.169 | <0.001 |  |
| Host N:P | -2.1E-09 | 1.78E-10 | -11.911 | <0.001 |  |
|  |  |  |  |  |  |
| Analysis of Variance: | | |  |  |  |
|  | DF | SS | MS | F | P |
| Regression | 1 | 1.66E-14 | 1.66E-14 | 141.874 | <0.001 |
| Residual | 10 | 1.17E-15 | 1.17E-16 |  |  |

Zoospore c vs. Host N:P

| zoospore c = 0.000957 - (0.0000155 * Host N:P) | | | | |  |
| --- | --- | --- | --- | --- | --- |
|  |  |  |  |  |  |
| N = 12 |  |  |  |  |  |
|  |  |  |  |  |  |
| R = 0.769 | Rsqr = 0.591 | Adj Rsqr = 0.550 | |  |  |
|  |  |  |  |  |  |
| Standard Error of Estimate = 0.000 | | | |  |  |
|  |  |  |  |  |  |
|  | Coefficient | Std. Error | t | P |  |
| Constant | 0.000957 | 0.000114 | 8.409 | <0.001 |  |
| Host N:P | -1.6E-05 | 4.07E-06 | -3.8 | 0.003 |  |
|  |  |  |  |  |  |
| Analysis of Variance: | | |  |  |  |
|  | DF | SS | MS | F | P |
| Regression | 1 | 8.83E-07 | 8.83E-07 | 14.437 | 0.003 |
| Residual | 10 | 6.12E-07 | 6.12E-08 |  |  |
| Total | 11 | 1.50E-06 | 1.36E-07 |  |  |

| zoospore n = 0.000214 - (0.00000374 * Host N:P) | | | | |  |
| --- | --- | --- | --- | --- | --- |
|  |  |  |  |  |  |
| N = 12 |  |  |  |  |  |
|  |  |  |  |  |  |
| R = 0.777 | Rsqr = 0.604 | Adj Rsqr = 0.564 | |  |  |
|  |  |  |  |  |  |
| Standard Error of Estimate = 0.000 | | | |  |  |
|  |  |  |  |  |  |
|  | Coefficient | Std. Error | t | P |  |
| Constant | 0.000214 | 2.68E-05 | 7.984 | <0.001 |  |
| Host N:P | -3.7E-06 | 9.57E-07 | -3.906 | 0.003 |  |
|  |  |  |  |  |  |
| Analysis of Variance: | | |  |  |  |
|  | DF | SS | MS | F | P |
| Regression | 1 | 5.17E-08 | 5.17E-08 | 15.256 | 0.003 |
| Residual | 10 | 3.39E-08 | 3.39E-09 |  |  |

Zoospore n vs. Host N:P

Zoospore p vs. Host N:P

| zoospore p = 0.0000170 - (0.000000334 * Host N:P) | | | | |  |
| --- | --- | --- | --- | --- | --- |
|  |  |  |  |  |  |
| N = 12 |  |  |  |  |  |
|  |  |  |  |  |  |
| R = 0.836 | Rsqr = 0.698 | Adj Rsqr = 0.668 | |  |  |
|  |  |  |  |  |  |
| Standard Error of Estimate = 0.000 | | | |  |  |
|  |  |  |  |  |  |
|  | Coefficient | Std. Error | t | P |  |
| Constant | 0.000017 | 1.94E-06 | 8.718 | <0.001 |  |
| Host N:P | -3.3E-07 | 6.95E-08 | -4.811 | <0.001 |  |
|  |  |  |  |  |  |
| Analysis of Variance: | | |  |  |  |
|  | DF | SS | MS | F | P |
| Regression | 1 | 4.13E-10 | 4.13E-10 | 23.143 | <0.001 |
| Residual | 10 | 1.79E-10 | 1.79E-11 |  |  |

Zoospore production rate vs. Host N:P

| zoopore production rate = 1.884 + (0.0202 * Host N:P) | | | | | |
| --- | --- | --- | --- | --- | --- |
|  |  |  |  |  |  |
| N = 12 |  |  |  |  |  |
|  |  |  |  |  |  |
| R = 0.611 | Rsqr = 0.373 | Adj Rsqr = 0.311 | |  |  |
|  |  |  |  |  |  |
| Standard Error of Estimate = 0.504 | | | |  |  |
|  |  |  |  |  |  |
|  | Coefficient | Std. Error | t | P |  |
| Constant | 1.884 | 0.232 | 8.131 | <0.001 |  |
| Host N:P | 0.0202 | 0.00828 | 2.44 | 0.035 |  |
|  |  |  |  |  |  |
| Analysis of Variance: | | |  |  |  |
|  | DF | SS | MS | F | P |
| Regression | 1 | 1.51E+00 | 1.51E+00 | 5.956 | 0.035 |
| Residual | 10 | 2.54E+00 | 2.54E-01 |  |  |

Zoospore production efficiency vs. Host N:P

| zoospore production efficiency = 0.00000218 + (0.0000000584 * Host N:P) | | | | | |
| --- | --- | --- | --- | --- | --- |
|  |  |  |  |  |  |
| N = 12 |  |  |  |  |  |
|  |  |  |  |  |  |
| R = 0.852 | Rsqr = 0.725 | Adj Rsqr = 0.698 | |  |  |
|  |  |  |  |  |  |
| Standard Error of Estimate = 0.000 | | | |  |  |
|  |  |  |  |  |  |
|  | Coefficient | Std. Error | t | P |  |
| Constant | 2.18E-06 | 3.18E-07 | 6.863 | <0.001 |  |
| Host N:P | 5.84E-08 | 1.14E-08 | 5.14 | <0.001 |  |
|  |  |  |  |  |  |
| Analysis of Variance: | | |  |  |  |
|  | DF | SS | MS | F | P |
| Regression | 1 | 1.26E-11 | 1.26E-11 | 26.417 | <0.001 |
| Residual | 10 | 4.78E-12 | 4.78E-13 |  |  |

| zoospore production efficiency = 0.00000529 - (0.00295 * zoospore c) | | | | | |
| --- | --- | --- | --- | --- | --- |
|  |  |  |  |  |  |
| N = 12 |  |  |  |  |  |
|  |  |  |  |  |  |
| R = 0.866 | Rsqr = 0.749 | Adj Rsqr = 0.724 | |  |  |
|  |  |  |  |  |  |
| Standard Error of Estimate = 0.000 | | | |  |  |
|  |  |  |  |  |  |
|  | Coefficient | Std. Error | t | P |  |
| Constant | 5.29E-06 | 3.85E-07 | 13.72 | <0.001 |  |
| zoospore c | -0.00295 | 0.00054 | -5.469 | <0.001 |  |
|  |  |  |  |  |  |
| Analysis of Variance: | | |  |  |  |
|  | DF | SS | MS | F | P |
| Regression | 1 | 1.30E-11 | 1.30E-11 | 29.906 | <0.001 |
| Residual | 10 | 4.36E-12 | 4.36E-13 |  |  |

Zoospore production efficiency vs. zoospore c

**
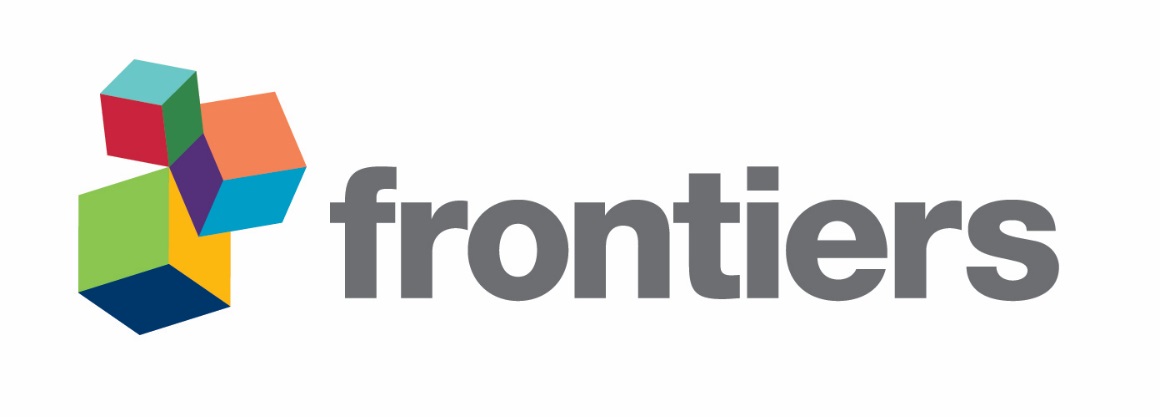
**
